# Determinants of infant feeding practices among mothers living with HIV attending prevention of mother to child transmission Clinic at Kiambu Level 4 hospital, Kenya: a cross-sectional study

**DOI:** 10.1186/s12937-019-0490-y

**Published:** 2019-11-02

**Authors:** Naureen Andare, Sophie Ochola, Peter Chege

**Affiliations:** School of Applied Human Sciences, Department of Food, Nutrition and Dietetics, Kenyatta University, Nairobi, P.O BOX 43844-00100 Kenya

**Keywords:** Determinants, Infant feeding practice, Mothers, HIV, Prevention of mother-to-child transmission

## Abstract

**Background:**

Human immunodeficiency virus infection and acquired immune deficiency syndrome is global pandemic with around 150,000 children infected with HIV in 2015. In Kenya, it is estimated that 11,000 children who are under 15 years old were infected. Safe infant feeding practices are the major important determinants of the prevention of mother to child transmission. The decision to breastfeed or not is considered a very tough choice for mothers living with HIV. This study assessed the infant feeding practices and its determinants among mothers living with HIV with infants 0–12 months old.

**Methods:**

This was a mixed methods cross-sectional study adopting qualitative and quantitative data collection procedures. A sample of 180 systematically selected mothers living with HIV with infants 0–12 months old attending prevention of mother to child transmission clinic participated in the study.

**Results:**

Exclusive breastfeeding rate was 71.4%, mixed feeding (18.2%) and replacement feeding (10.4%). Complementary feeding with continued breastfeeding rate was 63.1%. Similarly, qualitative results showed that exclusive breastfeeding was the most preferred feeding method by mothers living with HIV. Age (Adjusted Odds Ratio (AOR) 0.19; (95% Confidence Interval (CI) 0.41, 0.85; *p* = 0.030) and infant feeding practice knowledge (AOR 0.20; 95% CI 0.06, 0.64; *p* = 0.007) were determinants of exclusive breastfeeding. Education AOR 0.17; 95% CI 0.03, 0.85; *p* = 0.002) and occupation (AOR 3.91; 95% CI 1.24, 12.32; *p* = 0.020) were determinants of complementary feeding with continued breastfeeding.

**Conclusion:**

Exclusive breastfeeding is attainable in this population. However, poor infant feeding practice knowledge led to non-adherence to safe infant feeding practices such as exclusive breastfeeding. Socio-demographic factors such as age, education and occupation were established as determinants of infant feeding practices among mothers living with HIV. Ministry of Health should come up with strategies on infant feeding counseling that are aligned to a local context, to allow mothers to understand the importance of recommended infant feeding options for HIV-exposed infants.

## Background

Globally, it was estimated that 36.7 million people were living with Human Immunodeficiency Virus (HIV) in 2015 with about 150,000 new infections among children, 50% decline since 2010 [1]. In the same year, approximately 56,000 children were newly infected in Eastern and Southern Africa, a reduction of 66% since 2010. HIV can be transmitted during pregnancy, childbirth and breastfeeding from a mother living with HIV. The huge majority of infections in children (0–14 years) are caused by mother-to-child transmission (MTCT). The chance of MTCT during pregnancy is 15 to 45% without treatment. However, antiretroviral treatment (ART) may reduce the risk to less than 5% [2]. Prevention of mother-to-child transmission (PMTCT) services support safe childbirth practices and adequate infant feeding practices. The implementation of PMTCT services prevented around 1.4 million HIV infections among children between 2010 and 2018 [3].

In 2016, 740,000 women of reproductive age were diagnosed with HIV despite this significant progress and approximately half of 180,000 children were newly infected during breastfeeding [4]. World Health Organization (WHO) issued guidelines recommending that mothers living with HIV who were on ART could receive full support to breastfeed their infants exclusively for the first six months of life, then introduce appropriate complementary foods while continuing to breastfeed for at least 12 months and up to 24 months or longer [2].

Kenya is committed to eliminating HIV transmission from mother to child. Out of an estimated 79,000 eligible women, 59,000 accessed PMTCT services in 2015 (74% coverage) [5]. The number of newly infected children (0–14 years) dropped from 12,000 in 2010 to 6600 in 2015, largely due to PMTCT services. Kenya adopted the recommendations of the World Health Organization to provide immediate treatment for people with HIV diagnosis in 2015. As a result, about 940,000 adults and 60,000 children accessed ART in 2016 [5]. Recent statistics have shown that 68% of women in Kenya have accessed ART [6].

In 2015, Kiambu County contributed 1.1 and 6.0% of Kenya’s total new HIV infections among children and adults respectively, and the overall ART coverage was 92%. Approximately 1951 pregnant women living with HIV accessed PMTCT services in Kiambu County. Only 5.0% children were infected with HIV in 2015 in Kiambu, 60% reduction from 2013 which indicated an improvement in reducing mother to child transmission of HIV [5].

Irrespective of HIV status, 61.4% of Kenyan children below 6 months of age are exclusively breastfed [7]. Even though breastfeeding is a common practice in Kenya some mothers, regardless of their HIV status, prefer mixed feeding rather than exclusive breastfeeding. Decision on whether to breastfeed or not is considered as a complicated choice for mothers living with HIV [8, 9]. The challenges and difficulties faced by mothers living with HIV could have negative consequences in the effectiveness of the PMTCT services especially in locales where resources are inadequate [10]. The purpose of this study was to assess the infant feeding practices and its determinants among mothers living with HIV with infants 0–12 months old attending PMTCT clinic at the Kiambu Level 4 Hospital.

## Methods

### Study design, setting and period

The study adopted a cross-sectional study design with both qualitative and quantitative methods of data collection and analysis. It was conducted at the Ministry of Health Kiambu Level 4 Hospital in the PMTCT clinic. Some of the services offered here include antenatal care and PMTCT, infant feeding counseling, nutrition assessment and education. Kiambu is located in Kiambu County in Kenya. The County is adjacent to the northern border of Nairobi and has a total population of 1,623,208 [11]. Data collection was conducted for 3 months from January to March 2015.

### Study participants

The participants were 180 mothers living with HIV with infants 0–12 months old at the time of the study attending PMTCT clinic at the Kiambu Level 4 Hospital.

### Sample size and sampling techniques

The sample size for this study was calculated using Fischer et al. equation [12] at 95% confident interval based on 11% women of reproductive age with HIV in Central Province [8]. A further 20% was added in the sample to cater for non-response. The pre-determined sample of 180 respondents was systematically selected. An average PMTCT attendance was estimated to be 400 per month. The PMTCT services were available 3 days per week and therefore 33 women visited the clinic per day. Hence, a sampling interval of 3 was used to select 11 mothers per day. On a daily basis, first study subject was chosen randomly by balloting: selecting a number between one and the sampling interval (3). The remaining 10 women were systematically selected: selecting every third woman until the required 180 sample size was reached.

Additionally, two focus group discussions (FGDs) were conducted, each consisting of 10 mothers living with HIV with infants aged 0–12 months attending PMTCT clinic. For the FGDs, participants were randomly selected based on their HIV status and the age of their infants. Five key informant interviews were conducted with PMTCT Nurses, Counsellors, and Nutritionists. They were purposively sampled because they were knowledgeable about infant feeding practices in the context of HIV.

### Data collection tools and techniques

Quantitative data was collected using a researcher-administered semi-structured questionnaire. The questionnaire was designed to get information on the following: the demographic and socio-economic characteristics of mothers living with HIV; the infant feeding practices of mothers with regard to HIV; knowledge and attitude of mothers living with HIV relating to infant feeding practices. The FGD guide was used to collect information on perceptions towards infant feeding practices in the context of HIV. Key informant interviews were used to collect data of suggestions on how to improve infant feeding strategies in the PMTCT, preferred infant feeding choices and factors affecting mothers’ decisions on infant feeding.

The inclusion criterion was mothers aged 18 years and above who were living with HIV, with infants aged 0–12 months, attending the PMTCT clinic at the Kiambu Level 4 Hospital. The respondents who met the criteria were selected with the assistance of the nurse in-charge and mother-to-mother support group counselors upon agreeing to sign an informed and written consent. The researcher and the research assistants then administered the questionnaire in a private and secluded room to ensure confidentiality. Two FGDs, each containing a group of 10 mothers of reproductive age (18–49) living with HIV with infants 0–12 months, were conducted. Five key informant interviews (KIIs) were conducted with 2 PMTCT nurses, 1 counsellor and 2 nutritionists.

### Data analyses

Data was analyzed using Statistical Package for the Social Sciences (SPSS) for windows version 22. Descriptive statistics, including measures of central tendency (that is means and median) and dispersion as Standard Deviation (SD) and range, as well as frequencies and percentages was used to describe the study population and the infant feeding practices of the mothers. Bivariate analysis (ODDS RATIO) was conducted to establish associations between various variables and infant feeding practices. Variables which showed significant association were further subjected to multivariate analysis (logistic regression) to establish the determinants of infant feeding practices. A *p*-value of < 0.05 was used as the basis for statistical significance. QDA Miner (Qualitative Data Analysis) tool was used to analyze key informant interviews and focus group discussion transcripts. Qualitative data was coded into themes and the findings triangulated with quantitative data from the questionnaires.

### Infant feeding practice knowledge score analysis

Infant feeding practice knowledge of mothers living with HIV was assessed using eight questions in the following categories: MTCT of HIV during breastfeeding, feeding methods that reduce MTCT of HIV and use of ARTs in the prevention of MTCT. Each right response was granted a score of 1 and a wrong response granted a score of 0, giving aggregate score of 8. The overall knowledge score for each mother was dictated by the quantity of right responses. A mother was considered to have adequate infant feeding practice knowledge in the context of HIV if she scored correctly all the knowledge questions right [28].

## Results

### Demographic characteristics of the mothers

The median age for mothers interviewed was 30, with a range of 21–44 years. Majority of the mothers were married (68.9%) and had attained secondary level of education (43.3%). Most mothers were unemployed (42.8%) and depended on their spouses as household income providers (61.1%). Median parity was 2, with range 1 to 7 children. The average size of household was 4.2 ± 1.32 members (Table 1).

**Table 1:** Demographic characteristics of the mothers living with HIV with infants 0–12 months

Demographic information	Mothers with children < 6 months old (*n* = 77)	Mothers with children 6–12 months old (*n* = 103)	Total 0–12 months old (*n* = 180)
	n (%)	n (%)	n (%)
Age group (years)			
< 25	23 (29.9)	31 (30.1)	54 (30.0)
26–34	35 (45.5)	56 (54.4)	91 (50.6)
> 35	19 (24.7)	16 (15.5)	35 (19.4)
Marital status			
Married	56 (72.7)	68 (66)	124 (68.9)
Unmarried	21 (27.3)	35 (34)	56 (31.1)
Education level			
Tertiary	10 (13.0)	12 (11.7)	22 (12.2)
Secondary school	31 (40.3)	47 (45.6)	78 (43.3)
Primary school	31 (40.3)	39 (37.9)	70 (38.9)
No formal education	5 (6.5)	5 (4.9)	10 (5.6)
Occupation			
Employed	20 (26.0)	42 (40.8)	62 (34.4)
Unemployed	37 (48.1)	40 (38.8)	77 (42.8)
Casual labour	20 (26)	21 (20.4)	41 (22.8)
Household main provider			
Self	21 (27.3)	41 (39.8)	62 (34.4)
Spouse	51 (66.2)	59 (57.3)	110 (61.1)
Relatives	5 (6.5)	3 (2.9)	8 (4.4)
Median age (range)			30 (21–44)
Median parity (range)			2 (1–7)
Mean household size (SD)			4.2 ± 1.32

### Socio-economic characteristics of mothers

Nearly all the mothers (93.9%) purchased food from the market and 57.8% allocated 3000–6000 Kenyan Shillings (30–60 United States Dollar, 100 Kenyan shillings = 1 United States Dollar) of their monthly household income on food. The average Progress out of Poverty Index (PPI) [13] scores showed that 58.7% of the households lived below the national poverty line, while 41.3% lived above it. The components analyzed were ownership of household assets (television, radio, sofa, stove), dwelling unit characteristics (material of the roof and toilet facilities) and access to public services and resources (electricity).

### Infant feeding practices of mothers living with HIV

Breastfeeding was a common practice among mothers, and most of the children had been breastfed (91.7, 95% CI; 86.6–95.3), with 69.7% of mother having initiated timely breastfeeding (within 1 h of birth). The mean age for cessation for breastfeeding was 5.69 ± 3.75 SD, with some mothers stopping breastfeeding as early as 1 month of age. For mothers living with HIV with children less than 6 months old, exclusive breastfeeding was the most commonly practiced infant feeding (71.4, 95% CI; 60.4–80.3) followed by mixed feeding (18.2, 95% CI; 11.1–28.4) then replacement feeding (10.4, 95% CI; 5.2–19.5) (Table 2). Findings from key informant interviews corroborated the quantitative findings which showed that majority of the mothers living with HIV practices exclusive breastfeeding.

**Table 2:** Infant feeding practices of mothers living with HIV with infants 0–12 months

Infant feeding practices	*n* = 180		
	n	%	(95% CI)
Ever breastfed *(N = 180)*			
Yes	165	91.7	(86.6–95.3)
No	15	8.3	(4.7–13.4)
Timely initiation of breastfeeding *(N = 165)*			
Within 1 h	115	69.7	(62.3–76.2)
2–3 h	23	13.9	(9.0–20.2)
> 3 h but < 24 h	27	16.4	(11.1–22.9)
Feeding practices for children 0–5 months *(N = 77)*			
Exclusive breastfeeding	55	71.4	(60.4–80.3)
Replacement feeding	8	10.4	(11.1–28.4)
Mixed feeding	14	18.2	(5.2–19.5)
Feeding practices for children 6–12 months *(N = 103)*			
Complementary feeding with breast milk	65	63.1	(53.4–71.8)
Complementary feeding without breast milk	38	36.9	(27.6–47.0)

For mothers with children above 6 months old, most (63.1%) opted to continue breastfeeding after introducing complementary foods while 36.9% discontinued breastfeeding completely after introducing these foods (Table 2). The key informants stated that most mothers living with HIV adhere to complementary feeding methods with continued breastfeeding after 6 months.

### Infant feeding practice decision

Most mothers reported that a health care worker had influenced their current mode of infant feeding (48%), while 20% settled for their current mode of feeding because they felt that it was a risk for MTCT. Only 5% settled for their infant feeding choice in order to avoid stigmatization for their HIV status. Health workers’ role in influencing mothers’ infant feeding choice was also evident during the FGDs*;* mothers stated that they relied on health workers to support them in making choices of right infant feeding methods. Breastfeeding came out strong as a custom during FGDs and each mother was expected to breastfeed her child. A mother who chose not to breastfeed, gave the neighbours a clear indication of her HIV status, leading to stigmatization.

### Infant feeding practice knowledge

Most mothers were aware that breast milk prevented childhood illnesses (86.1%). They demonstrated understanding of the use of ART in the prevention of MTCT during breastfeeding (90.0%). With regard to continued breastfeeding, most mothers living with HIV showed awareness on continued breastfeeding up to 12 months, when both mother and infant were on ART (71.7%). Mothers understood that exclusive breastfeeding for 6 months was appropriate for all infants (86.7%) and indicated that mixed feeding in the first 6 months of life increased the risk of transmission of HIV from mother to the baby (75.6%).

More than half of the mothers (53.9%) had adequate knowledge on infant feeding practices having scored all the correct responses in knowledge. The study findings showed that the overall mean knowledge score on infant feeding practices was 6.36 ± 1.43 SD. Mothers’ knowledge scores were further subjected to one-way ANOVA analysis. The results showed a significant relationship between knowledge and infant feeding practices for children less than 6 months old (Table 3). Mothers who practiced mixed feeding had the lowest knowledge score on infant feeding in the context of HIV. This indicates that poor knowledge among mothers led to non-adherence to recommended feeding practices.

**Table 3:** Comparison between infant feeding practice knowledge scores and infant feeding practices of mothers living with HIV

Infant feeding practices	n	Mean knowledge scores (95% CI)	SD	df	f	ANOVA *p*-value
Children 0–5 months				2	4.36	0.016*
Exclusive breastfeeding	55	6.47 (6.17–6.78)	1.14			
Replacement feeding	8	6.63 (5.86–7.39)	0.92			
Mixed feeding	14	5.50 (4.76–6.24)	1.29			
Total	77	6.31 (6.04–6.58)	1.19			
Children 6–12 months				1	0.30	0.587
Complementary feeding breast milk	65	6.32 (5.95–6.70)	1.50			
Complementary feeding with no breast milk	38	6.50 (5.93–7.07)	1.74			
Total	103	6.39 (6.08–6.70)	1.59			

*Significance at p < 0.05

### Determinants of infant feeding practices among mothers

The determinants of infant feeding practices of mothers living with HIV are presented in Tables 4 and 5. Younger mothers had less chances of practising exclusive breastfeeding (AOR 0.19; 95% CI 0.41, 0.85; *p* = 0.030) compared to older mothers and mothers aged 26–34 years who were more likely to practice exclusive breastfeeding. However, this was not statistically significant (Table 4). Mothers with adequate infant feeding practice knowledge had greater chances of practising exclusive breastfeeding (AOR 0.20; 95% CI 0.06, 0.64; *p* = 0.007).

**Table 4:** Determinants of exclusive breastfeeding practices among mothers living with HIV with children 0–5 months

Variables	Exclusive breastfeeding				
	n (%)	COR (95% CI)	*P* value	AOR (95% CI)	*P* value
Age group (years					
< 25	23 (29.9)	0.24 (0.06, 0.97)	0.044*	0.19 (0.41, 0.85)	0.030*
26–34	35 (45.5)	1.29 (0.32, 5.28)		1.72 (0.32, 9.23)	
> 35	19 (24.7)	1		1	
Marital status					
Married	56 (72.7)	1.38 (0.46, 4.04)		1.46 (0.40, 5.34)	
Unmarried	21 (27.3)	1		1	
Education level					
No formal education	5 (6.5)	0.11 (0.01, 1.41)		0.11 (0.01, 1.39)	
Primary school	31 (40.3)	1.05 (0.22, 4.98)		1.04 (0.22, 5.01)	
Secondary school	31 (40.3)	1.79 (0.35, 9.02)		2.01 (0.39, 10.53)	
Tertiary	10 (13.0)	1		1	
Occupation					
Employed	20 (26.0)	1.04 (0.29, 3.66)		1.36 (0.28, 6.71)	
Unemployed	37 (48.1)	0.50 (0.13, 1.93)		2.17 (0.55, 8.58)	
Casual labour	20 (26)	1		1	
Infant feeding practice knowledge					
Adequate	39 (50.6)	1.82 (0.06, 0.57)	0.003*	0.20 (0.06, 0.64)	0.007*
Poor	38 (49.4)	1		1	

*AOR* Adjusted Odds Ratio*, COR* Crude Odds Ratio*, CI* Confidence Interval*.*

*Statistically significant at p < 0.05. Adjusted for socioeconomic and demographic characteristics (age, marital status, education level and occupation) and infant feeding practice knowledge).

**Table 5:** Determinants of complementary feeding with breastfeeding practices among mothers living with HIV with children 6–12 months

Variables	Complementary feeding with breastfeeding				
	n (%)	COR (95% CI)	*P* value	AOR (95% CI)	*P* value
Age group (years					
< 25	31 (30.1)	0.96 (0.24, 3.84)		0.87 (0.18, 4.26)	
26–34	56 (54.4)	0.39 (0.11, 1.34)		0.33 (0.08, 1.41)	
> 35	16 (15.5)	1		1	
Marital status					
Married	68 (66)	1.75 (0.72, 4.21)		1.89 (0.68, 5.29)	
Unmarried	35 (34)	1		1	
Education level					
No formal education	5 (4.9)	0.83 (0.01, 1.07)		0.56 (0.03, 0.96)	
Primary school	39 (37.9)	0.86 (0.02, 0.39)		0.46 (0.08, 0.28)	
Secondary school	47 (45.6)	0.25 (0.05, 1.03)	0.042*	0.17 (0.03, 0.85)	0.002*
Tertiary	12 (11.7)	1		1	
Occupation					
Employed	42 (40.8)	1.56 (0.69, 3.55)		2.06 (0.69, 6.15)	
Unemployed	40 (38.8)	4.00 (1.30, 12.2)	0.015*	3.91 (1.24, 12.32)	0.020*
Casual labour	21 (20.4)	1		1	
Infant feeding practice knowledge					
Adequate	58 (56.3)	1.75 (0.73, 4.21)		1.44 (0.59, 3.47)	
Poor	45 (43.7)	1		1	

*AOR* Adjusted Odds Ratio*, COR* Crude Odds Ratio*, CI* Confidence Interval*.*

*Statistically significant at p < 0.05. Adjusted for socioeconomic and demographic characteristics (age, marital status, education level and occupation) and infant feeding practice knowledge).

Mothers with secondary education level had less chances of practising complementary feeding with continued breastfeeding as compared to mothers with lower levels of education (AOR 0.17; 95% CI 0.03, 0.85; *p* = 0.002). Mothers who were unemployed had greater odds of practising complementary feeding with continued breastfeeding in comparison to those who worked (AOR 3.91; 95% CI 1.24, 12.32; *p* = 0.020) (Table 5). Due to lack of adequate sample size and precision of estimates, replacement feeding, mixed feeding and complementary feeding with no breast milk were not subjected to regression analysis, therefore not presented in the results.

## Discussions

The findings of this study showed that breastfeeding was acceptable, with majority of the mothers having initiated it. Of those who initiated breastfeeding, majority did so timely within 1 h after delivery. The same results were also reported by a study conducted in Homa-Bay, Kenya, on feeding practices and nutritional status of HIV exposed infants where majority of mothers initiate breastfeeding at birth [14]. The duration of breastfeeding diminished as the age of the child increased. The median age of discontinuity of breastfeeding in this study was 5.7 months, a rate lower than the Nairobi Province at 15.0 months [8]. This implied that many infants were not receiving breast milk as recommended and were therefore most likely not to get the health benefits of continued breastfeeding for the advocated period of at least 12 months and up to 24 months or longer [2]. A likely explanation for low span of breastfeeding in this study could be due to the trepidation of mothers to infect their children through transmission of HIV amid breastfeeding.

Majority of the mothers living with HIV in this study adhered to the recommended guidelines of exclusive breastfeeding up to 6 months. The finding of this study was similar to the finding of a study conducted in Bomet, Kenya [15]. The same results were observed in a study conducted in Kisumu, Kenya within a PMTCT [16]. Due to the improved PMTCT infant feeding counseling services, mothers had understood the importance of exclusive breastfeeding in the context of HIV. This implies that exclusive breastfeeding was an achievable infant feeding practice in Kenya.

In this study, only 10.4% of the mothers practised replacement feeding. This finding was in agreement with those from the findings of a study done in rural parts of Kisumu County, Kenya, where only 10% of the mothers living with HIV practised replacement feeding [17]. The low rate of replacement feeding practices was due to low socio economic status of mothers. Therefore, their inability to afford and maintain formula food as reported by mothers in FGDs and health workers in KIIs.

The proportion of mothers practising mixed feeding in this study was (18.2%) comparatively higher than that reported from a study conducted at Kitale District Hospital, Kenya [18], where mixed feeding was reported at 14.0%. The rate of mixed feeding in this study was also higher than in the study from Ethiopia (15.3%) [19]. A likely explanation for the findings in this study was because mothers perceived that breast milk was insufficient for the baby.

In this study, majority (63.1%) of the mothers practised complementary feeding with breast milk for children older than 6 months. Even though this was a positive finding, a large number of children under the age of 12 months were not receiving adequate nutrients from breast milk. One study from Tanzania on breastfeeding and complementary feeding practices among HIV-exposed infants revealed that majority of infants older than 6 months no longer received breast milk with only 14% infants older than 6 months still being breastfed [20]. These results implied that mothers perceived prolonged breastfeeding as a risk of HIV transmission to their children.

The findings showed that the mothers’ reason for choosing replacement feeding method was that they feared transmitting HIV to their infants. A study conducted in South Africa on effect of the HIV epidemic on infant feeding was in agreement with this finding [10]. Similarly, some of the commonest reasons cited in a study in Ethiopia for non-initiation of breastfeeding was fear of baby getting ill [19]. This was probably because some mothers still doubted the safety of breast milk for their HIV-exposed babies. Mothers also echoed these perspectives during focus group discussions in this study*.*

Majority of mothers demonstrated awareness on recommended infant feeding options in the context of HIV. This finding was similar with findings from a study conducted in Ethiopia on determinants of infant feeding practices of mothers living with HIV in public health institutions where majority of mothers (88.1%) had sufficient knowledge on safe infant feeding options [21]. In this study, most mothers demonstrated good understanding of the use of ART in the prevention of MTCT and that mothers could continue breastfeeding their infants up to 12 months. The same results were observed in a study conducted in Zambia [22].

Results revealed that age and knowledge were determinants of exclusive breastfeeding among mothers living with HIV. Younger mothers had less chances of practising exclusive breastfeeding compared to older mothers. The findings of this study were in contrast with findings from a study conducted in Ethiopia where mothers’ age had no association with breastfeeding practices [23]. Similarly, a cross-sectional study conducted in Uganda revealed that age was not significantly associated with exclusive breastfeeding [24]. Mothers with adequate knowledge had greater chances of practising exclusive breastfeeding. The finding was comparable to those of a study conducted in Tanzania [9].

Mother’s occupation was a determinant of complementary feeding with continued breastfeeding. Mothers who were unemployed had greater odds of practicing complementary feeding with continued breastfeeding. Similarly, a study conducted in Ethiopia on infant feeding practice and associated factors of mothers living with HIV attending PMTCT revealed that unemployed mothers’ homemakers were more likely to practise recommended infant feeding practices [25]. Education level was a determinant of infant feeding practices by the mothers. Mothers with low levels of education were more likely to practise complementary feeding with continued breastfeeding as compared to mothers with high levels of education. This finding concurred with findings from Lars and Leshabari [26, 27] which suggested that mothers with high levels of education breastfed their infants for a shorter time as compared to mothers with low levels of education.

### Limitations of the study

The study’s sample consisted of mothers attending PMTCT clinic at the time, which may not have been representative of the general population of mothers living with HIV with infants aged 0–12 months at Kiambu Level 4 Hospital. The findings could therefore, only be generalized with studies conducted in similar circumstances.

## Conclusions

Even though exclusive breastfeeding was a common practice among mothers living with HIV, a proportion of mothers still practised non-recommended infant feeding options such as mixed feeding, thus exposing their infants to MTCT. Knowledge was a determinant of recommended infant feeding practices, implying that mothers with poor knowledge did not adhere to safe infant feeding practices and were, therefore, at a high risk of MTCT. Additionally, socio-demographic factors such as age, education and occupation were determinants of infant feeding practices among mothers living with HIV. We recommend that the Ministry of Health comes up with strategies to ensure that infant feeding counseling and nutrition education received by mothers were aligned to local context. This will not only promote adherence to recommended infant feeding practices but will also encourage mothers living with HIV to make informed infant feeding choices.

## Data Availability

Data supporting the findings of this article is provided in the document.

## Abbreviations

AIDS: Acquired Immunodeficiency Syndrome
AOR: Adjusted Odds Ratio
ART: Antiretroviral Treatment
COR: Crude Odds Ratio
FGD: Focus Group Discussion
HIV: Human Immunodeficiency Virus
KII: Key Informant Interview
MTCT: Mother to Child Transmission
NACOSTI: National Council for Science and Technology and Innovation
PMTCT: Prevention of Mother to Child Transmission
PPI: Progress out of Poverty Index
QDA: Qualitative Data Analysis
SD: Standard Deviation
SPSS: Statistical Package for Social Sciences
WHO: World Health Organization
